# Ventilatory demand and dynamic hyperinflation induced during ADL-based tests in Chronic Obstructive Pulmonary Disease patients

**DOI:** 10.1590/bjpt-rbf.2014.0170

**Published:** 2016-06-16

**Authors:** Karoliny dos Santos, Aline A. Gulart, Anelise B. Munari, Manuela Karloh, Anamaria F. Mayer

**Affiliations:** 1 Núcleo de Assistência, Ensino e Pesquisa em Reabilitação Pulmonar, Universidade do Estado de Santa Catarina (UDESC), Florianópolis, SC, Brazil; 2 Programa de Pós-graduação em Fisioterapia, Centro de Ciências da Saúde e do Esporte (CEFID), UDESC, Florianópolis, SC, Brazil

**Keywords:** pulmonary disease, chronic obstructive, outcome assessment, activities of daily living, upper extremity, rehabilitation

## Abstract

**Background:**

Airflow limitation frequently leads to the interruption of activities of daily living (ADL) in patients with Chronic Obstructive Pulmonary Disease (COPD). These patients commonly show absence of ventilatory reserve, reduced inspiratory reserve volume, and dynamic hyperinflation (DH).

**Objective:**

To investigate ventilatory response and DH induced by three ADL-based protocols in COPD patients and compare them to healthy subjects.

**Method:**

Cross-sectional study. COPD group: 23 patients (65±6 years, FEV_1_ 37.2±15.4%pred); control group: 14 healthy subjects (64±4 years) matched for age, sex, and body mass index. Both groups performed all three tests: Glittre-ADL test; an activity test that involved moving objects on a shelf (T_SHELF_); and a modified shelf protocol isolating activity with upper limbs (T_SHELF-M_). Ventilatory response and inspiratory capacity were evaluated.

**Results:**

Baseline ventilatory variables were similar between groups (p>0.05). The ventilatory demand increased and the inspiratory capacity decreased significantly at the end of the tests in the COPD group. Ventilatory demand and DH were higher (p<0.05) in the T_SHELF_ than in the T_SHELF–M_ in the COPD group (p<0.05). There were no differences in DH between the three tests in the control group (p>0.05) and ventilatory demand increased at the end of the tests (p<0.05) but to a lower extent than the COPD group.

**Conclusion:**

The T_SHELF_ induces similar ventilatory responses to the Glittre-ADL test in COPD patients with higher ventilatory demand and DH. In contrast, the ventilatory response was attenuated in the T_SHELF-M_, suggesting that squatting and bending down during the Glittre-ADL test could trigger significant ventilatory overload.

## BULLET POINTS

- The Glittre-ADL test and the isolated shelf test cause similar ventilatory responses.
- The shelf test is probably the main cause of ventilatory overload during the Glittre-ADL test.
- Squatting and bending down could trigger ventilatory overload during the Glittre-ADL test.

## Introduction

Ventilatory limitation is frequently the main cause of interruption of exercise^1^ and activities of daily living (ADL)^2^ ^,^ ^3^ in patients with chronic obstructive pulmonary disease (COPD). Their ventilation nears maximal ventilatory capacity during physical activities, while cardiovascular and other physiological functions stay below maximum capacity^2^. Lack of ventilatory reserve, reduction in inspiratory reserve volume, and dynamic hyperinflation (DH) are commonly considered limiting factors during ADL in COPD patients^2^ ^,^ ^4^. The ventilatory response pattern, however, seems to be different between activities performed with lower and upper limbs^5^ ^,^ ^6^. When performed under the same metabolic demand, activities with upper extremities reach higher pulmonary ventilation and the majority of patients develop DH^7^. Nevertheless, the contribution of upper limb tasks to the development of DH and consequent limitation during ADL in COPD patients remains unclear.

The Glittre-ADL test is a multiple-task test that assesses functional status related to ADL in stable^8^ ^-^ ^11^ and hospitalized^12^ ^,^ ^13^ patients with respiratory disease. It includes activities that require rising from a seated position, activities with lower and upper extremities, as well as walking^8^. Recently, Karloh et al.^9^ compared the physiological response induced by the Glittre-ADL test with the six-minute walk test in COPD patients and observed that the peak oxygen consumption was approximately 7% higher (p<0.05) in the Glittre-ADL test, while the cardiovascular and ventilatory responses were similar between tests. During the Glittre-ADL test COPD, patients spend 50 to 65% of the test duration on squatting, bending down, standing, and moving objects on and off a shelf, which is often reported as the most exhausting activity of the test^9^. Based on this, it is hypothesized that these activities may have a significant influence on the increase in oxygen uptake and ventilatory demand (minute ventilation to maximum voluntary ventilation ratio - VE/MVV) during the Glittre-ADL test in these patients. Even though the physiological responses of the Glittre-ADL test have already been described^9^, the analysis of ventilatory variables has yet to be explored in detail in these patients and compared to healthy individuals. Furthermore, it is unknown how the activities performed with the shelf contribute to the ventilatory adjustments in COPD patients during the Glittre-ADL test. These are important points given that the activities performed with the shelf simulate common ADL that involve different mechanisms of limitation in COPD patients. In addition, previous studies have hypothesized that the upper-limb activation in the shelf activities may be responsible for thoracoabdominal asynchrony and consequently DH during the test^8^ ^,^ ^9^, especially in severe patients^8^. Thus, the objective of the study was to investigate ventilatory responses and DH during three ADL-based tests in COPD patients and compare their responses with those of healthy subjects.

## Method

### Subjects

Twenty-three subjects with COPD were recruited from respiratory clinics in Florianópolis, SC, Brazil, to take part in the study. The control group was composed of 14 healthy subjects matched with the COPD group (COPDG) for gender, age, and body mass index. The study was approved by the Human Research Ethics Committee of Universidade **do Estado** de Santa Catarina (UDESC), Florianópolis, SC, Brazil (CAAE: 07397212.3.0000.0118), and all participants signed an informed consent form.

### Inclusion and exclusion criteria

Patients who met the following criteria were included in the COPDG: stages 2-4 according to the Global Initiative for Chronic Obstructive Lung Disease (GOLD) guidelines^14^, smoking history >20 pack-years, former smokers (smoking cessation history longer than 6 months), age ≥40 years-old, and clinical stability in the past month prior to enrollment in the study. The exclusion criteria were long-term oxygen therapy, cardiomyopathy, musculoskeletal disorders, cancer, tuberculosis, asthma, use of orthopedic prosthesis, inability to perform any test required in the study, exacerbation of the disease during the study, or exercise rehabilitation program completed within one year from enrollment in the study.

The control group had normal lung function (forced expiratory volume in first second and forced vital capacity ratio>0.7; forced expiratory volume in first second >80% predicted and forced vital capacity >80% predicted) and were sedentary (“low” score on the International Physical Activity Questionnaire–IPAQ)^15^. Subjects who presented other related diseases or who were unable to perform any test of the study protocol were excluded.

### Protocol

This was a cross-sectional study completed on two different days. On the first day, participants completed anthropometric and pulmonary function evaluation and randomly performed two different tests involving shelf activities. A 30-min resting time between tests was established in order to ensure that heart rate and pulse oximetry returned to baseline values. On the second day, participants performed two Glittre-ADL tests with the same resting period between them. Each test was previously explained and demonstrated to the participants and no verbal encouragement was given. COPD patients received bronchodilator (albuterol 400 mcg) 15 minutes prior to all tests on both days.

### Pulmonary function

Pulmonary function testing was performed with the EasyOne spirometer (NDD Medical Technologies Inc., Zurich, Switzerland), which was calibrated on every evaluation day. The reproducibility and acceptability criteria were based on ATS/ERS^16^ guidelines and the reference values were based on the equation of Pereira et al.^17^.

### Glittre-ADL test

The Glittre-ADL test consists of completing a circuit while carrying a weighted backpack (2.5 kg for women, 5.0 kg for men) as follows. From a sitting position, the subject stands up and walks along a flat 10-m course in the middle of which there is a two-step staircase (each step 17 cm high × 27 cm deep) to be traversed. After completing the last 5 m, the subject comes to a shelf containing three objects, each weighing 1 kg positioned on the top shelf (shoulder height), and moves them one by one to the bottom shelf (waist height) and then to the floor. The objects are then returned to the bottom shelf and finally to the top shelf. The subject then walks back along the circuit, climbing and descending the stairs, until reaching the starting point (chair), then sits down and immediately begins the next lap^8^. The patients were instructed to complete five laps on this circuit as quickly as possible. Two tests were performed and the fastest test was selected for analysis.

### Shelf test (T_SHELF_)

**Figure 1:**
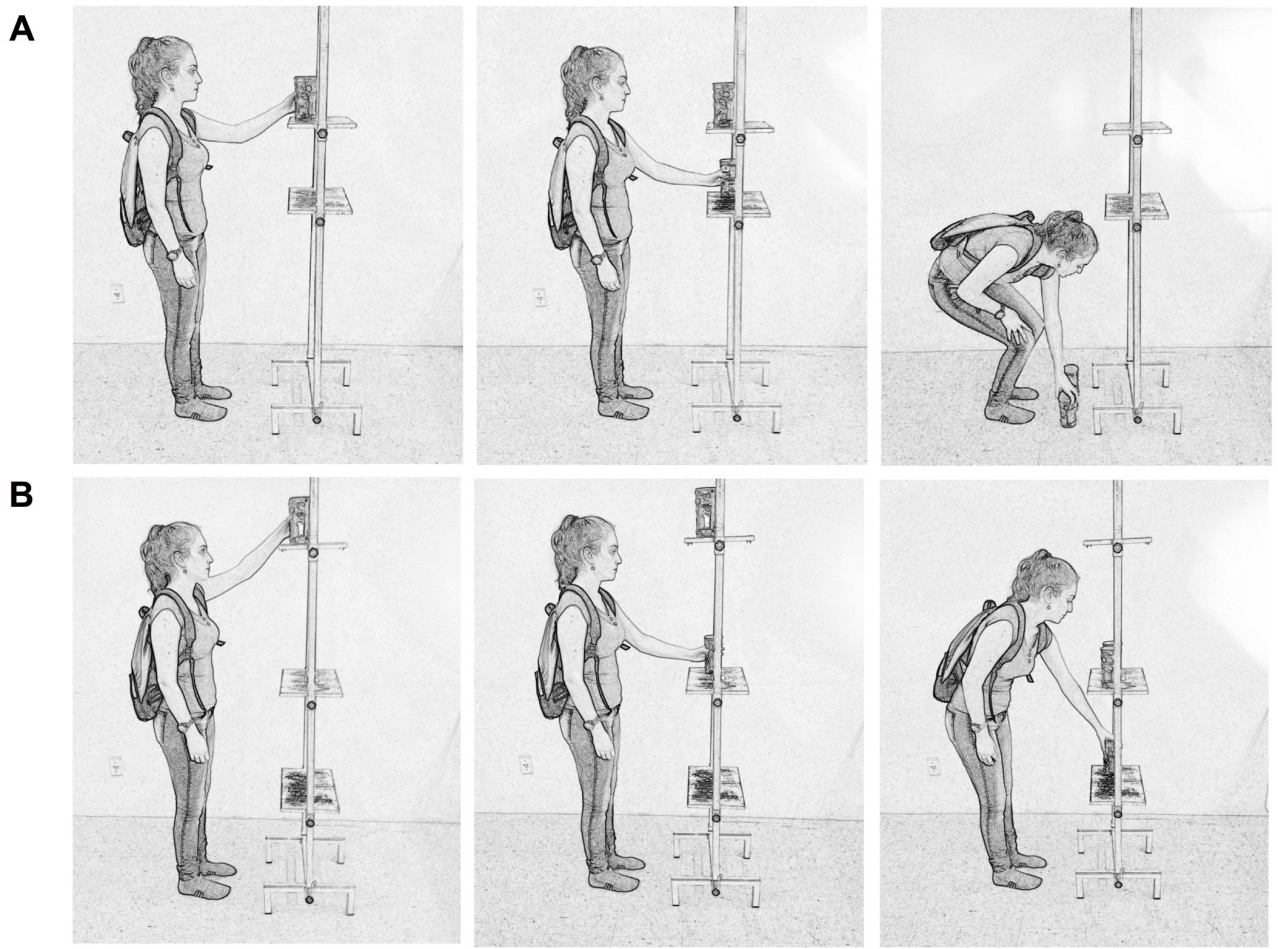
(A) Shelf test; (B) Modified shelf test.

The development of this test was based on the activities of the Glittre-ADL test performed in front of the shelf. The height of the shelves, the weight of the objects, and the backpack were the same used in the Glittre-ADL test^8^. One lap consisted of moving the objects one by one from the top shelf to the bottom shelf and to the floor, then returning them to the bottom shelf and finally to the top shelf (Figure 1A). Participants should complete five laps in the shortest time possible.

### Modified shelf test (T_SHELF-M_)

This test was similar to the T_SHELF_ with modifications to the height of shelves and backpack weight. The 1 kg objects were moved among three shelves: the top shelf at eye level, the intermediate at waist level, and the bottom at the distal apex of the patellae. One lap was considered as moving the objects from the top shelf to the intermediate shelf and the bottom shelf, then from the bottom to the intermediate and back to the top shelf (Figure 1B). The participants received instructions to complete five laps within the shortest time possible. All participants carried a 2.5 kg backpack, independent of gender. The changes were made to isolate the use of upper limbs, reducing the effects of squatting and bending down, as well as the overload caused by the backpack weight carried by the male subjects.

### Inspiratory capacity

Prior to and immediately after completing the test, a slow vital capacity test was performed according to ATS/ERS guidelines^16^. The measurements were completed using the EasyOne portable spirometer (NDD Medical Technologies Inc., Zurich, Switzerland) with patients in the seated position. A minimum of three and maximum of eight attempts were allowed and the reproducibility was defined as a difference of less than 5% or 150 ml between two maneuvers^1^ ^,^ ^18^ ^,^ ^19^. The highest reproducible values were considered for analysis. DH was defined as a decrease in inspiratory capacity of at least 10% and/or 150ml compared to baseline^1^. After completing the tests, all participants performed the inspiratory capacity post-test maneuvers within 5 minutes^6^.

### Ventilatory response

During the Glittre-ADL test, T_SHELF_, and T_SHELF-M_, participants used a portable spirometer (Spiropalm 6MWT; Cosmed, Rome, Italy) consisting of a data capture unit carried on a belt adjusted to the waist and a flow meter attached to a facial mask. The equipment was calibrated according to manufacturer’s recommendations. The following variables were measured during the tests: minute ventilation, respiratory rate, expiratory time, inspiratory-to-total cycle time ratio, and VE/MVV. For data analysis, the mean of the final 15 seconds of each lap of the Glittre-ADL test and the final 15 seconds of the T_SHELF_ and T_SHELF-M_ were considered. Maximum voluntary ventilation was calculated based on the equation: forced expiratory volume in first second × 37.5^20^.

### International Physical Activity Questionnaire (IPAQ)

The IPAQ short form was administered to the control group as an interview to evaluate the physical activity level of the participants^15^. The IPAQ includes questions about frequency and duration of walking, moderate activities, and vigorous activities and classifies the level of physical activity as low, moderate, or high^15^. Only participants with “low” score were considered eligible for this study.

### Sample Size

To calculate the sample size for the present study, we used data from a pilot study and from a study that evaluated VE/MVV induced by the Glittre-ADL test^9^. Based on a minimum increase of 20% in VE/MVV and decrease of 150mL in inspiratory capacity between the Glittre-ADL test, T_SHELF_, and T_SHELF-M_ and considering an α=0.05, a power of 80%, and a dropout rate of 10%, the estimated sample size was 22 patients.

### Data analysis

The data were reported as mean and standard deviation (SD). The Shapiro-Wilk test was used to analyze data normality. An unpaired t-test or Mann-Whitney U test was used to compare the performance, ventilatory response, and inspiratory capacity during activities between the COPDG and control group. One-way ANOVA followed by Tukey’s post hoc test were used to analyze the ventilatory variables between activities within each group. Statistical significance was set at p<0.05. Data analysis was performed with SPSS 21.0.

## Results

Of the 26 patients selected for the study, three were excluded. Two did not reach the spirometric criteria and one was unable to perform the tests wearing the facial mask. Out of the 17 healthy volunteers, three were excluded: one for not finalizing the protocol, one for failing to reproduce the inspiratory capacity maneuver and one for not reaching the spirometric criteria. In total, 23 COPD patients (16 males) and 14 healthy subjects (9 males) were included in this study between June 2013 and April 2014. Groups were similar in age, weight, height, and body mass index. Demographic and pulmonary function data are shown in Table 1.

**Table 1:** Characteristics of the study groups.

	**COPD Group** **(n=23)**	**Control Group** **(n=14)**	**p**
Age, years	65.7±6.61	64.2±4.57	0.467
Weight, kg	70.0±13.1	74.7±9.12	0.240
Height, m	1.64±0.06	1.66±0.09	0.521
BMI, kg/m^2^	25.7±4.50	26.9±2.32	0.289
Smoking history, pack-years	61.1±31.4	4.64±8.42	0.001
GOLD 2, *n*	7		
GOLD 3, *n*	7		
GOLD 4, *n*	9		
FEV_1_/FCV	0.47±0.10	0.80±0.05	0.001
FEV_1_. L	1.08±0.45	2.70±0.60	0.001
FEV_1_, %pred	37.2±15.4	90.7±9.50	0.001
FVC, L	2.23±0.64	3.38±0.70	0.001
FVC, %pred	60.5±16.6	88.7±8.84	0.001
MVVpred. L/min	40.6±17.1	101±22.6	0.001

Results are presented as mean±standard deviation. GOLD: Global Initiative for Chronic Obstructive Lung Disease; BMI: body mass index; FEV_1_: forced expiratory volume in first second; FVC: forced vital capacity; MVV: maximal voluntary ventilation; %pred: % predicted.

### Ventilatory response

**Figure 2:**
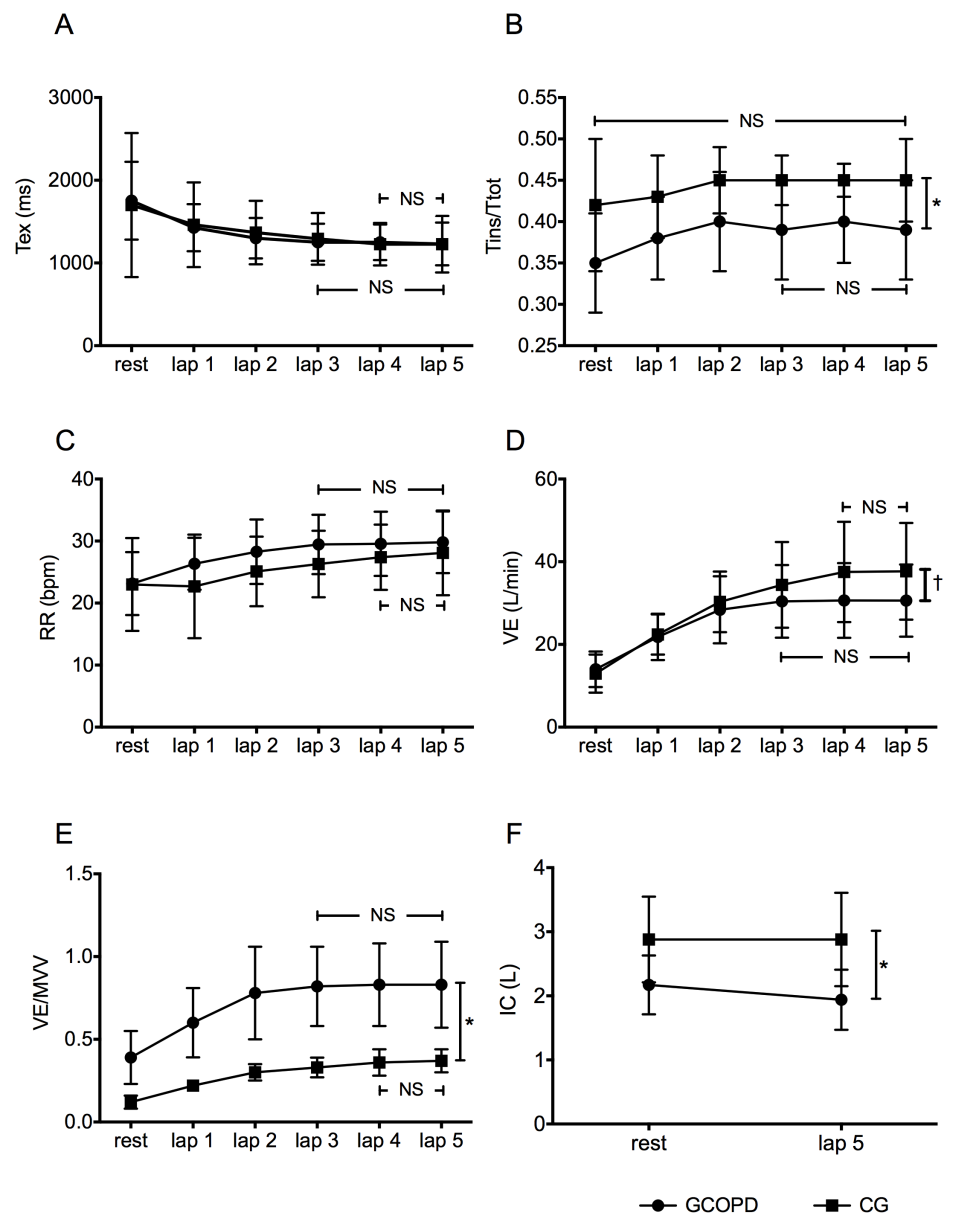
Ventilatory responses during the Glittre-ADL test in the COPD group (circles) and control group (squares) at rest and on each lap test. Average data and standard deviation: (A) expiratory time; (B) inspiratory time/total time; (C) respiratory rate; (D) minute ventilation; (E) ventilatory demand; (F) inspiratory capacity. * p<0.005; †: p<0.05; NS: not significant.

All variables showed significant changes compared with baseline values in both groups. The ventilatory variables in the COPDG and control group are presented in Table 2 and Figure 2. Both groups had similar ventilatory conditions at baseline for respiratory rate, minute ventilation, expiratory time, and inspiratory-to-total cycle time ratio. The COPDG had significantly lower baseline inspiratory capacity (p=0.003) and higher VE/MVV (p=0.001). In addition, the COPDG showed a tendency to stabilize respiratory rate, expiratory time, and minute ventilation on the second lap of the Glittre-ADL test (Figure 2).

**Table 2:** Behavior of ventilatory variables during the Glittre-ADL test, T_SHELF_, and T_SHELF-M_ in both groups.

	**Glittre-ADL test**		**T_SHELF_**		**T_SHELF-M_**	
	**∆**		**∆**		**∆**	
	**COPDG**	**CG**	**COPDG**	**CG**	**COPDG**	**CG**
Time, min	4.92±2.62‡ §	2.81±0.30‡ §	2.01±0.60	1.48±0.18	1.73±0.29	1.38±0.18
IC, L	0.22±0.16†	0.00±0.14	0.24±0.20† #	–0.05±0.16	0.11±0.16†	0.07±0.20
T_ex,_ ms	–523±445	–473±734	–406±502	–389±523	–280±475	–198±513
T_ins_/T_tot_ rate	0.03±0.06§	0.03±0.09	0.00±0.07#	–0.01±0.10	–0.15±0.08†	0.05±0.09
VE, L/min	16.6±6.26† §	24.7±9.23§	13.9±6.72† #	14.7±6.36#	9.03±4.22	7.11±2.62
RR, bpm	6.65±4.61	5.10±5.15	6.02±6.02*	1.75±5.98	4.31±4.66	1.38±5.15
VE/MVV	0.43±0.14† §	0.24±0.05§ ‡	0.37±0.14† #	0.14±0.05#	0.24±0.10†	0.07±0.02

Results are presented as mean ± standard deviation. **∆**: change (final – rest values); CG: control group; min: minutes; IC: inspiratory capacity; L: liters; ms: milliseconds; T_ex_: expiratory time; T_ins_: inspiratory time; T_tot_: total respiratory cycle; mL: milliliters; VE: minute ventilation; L/min: liters per minute; RR: respiratory rate; bpm: breaths per minute; MVV: maximal voluntary ventilation.

* p<0.01 COPD *vs.* Control;

† p<0.05 COPD *vs.* Control;

‡ p<0.05 *post ho*c Glittre-ADL test *vs.* T_SHELF_;

§ p<0.05 *post hoc* Glittre-ADL test *vs.* T_SHELF-M_;

# p<0.05 *post hoc* T_SHELF_ *vs.* T_SHELF-M_.

**Figure 3:**
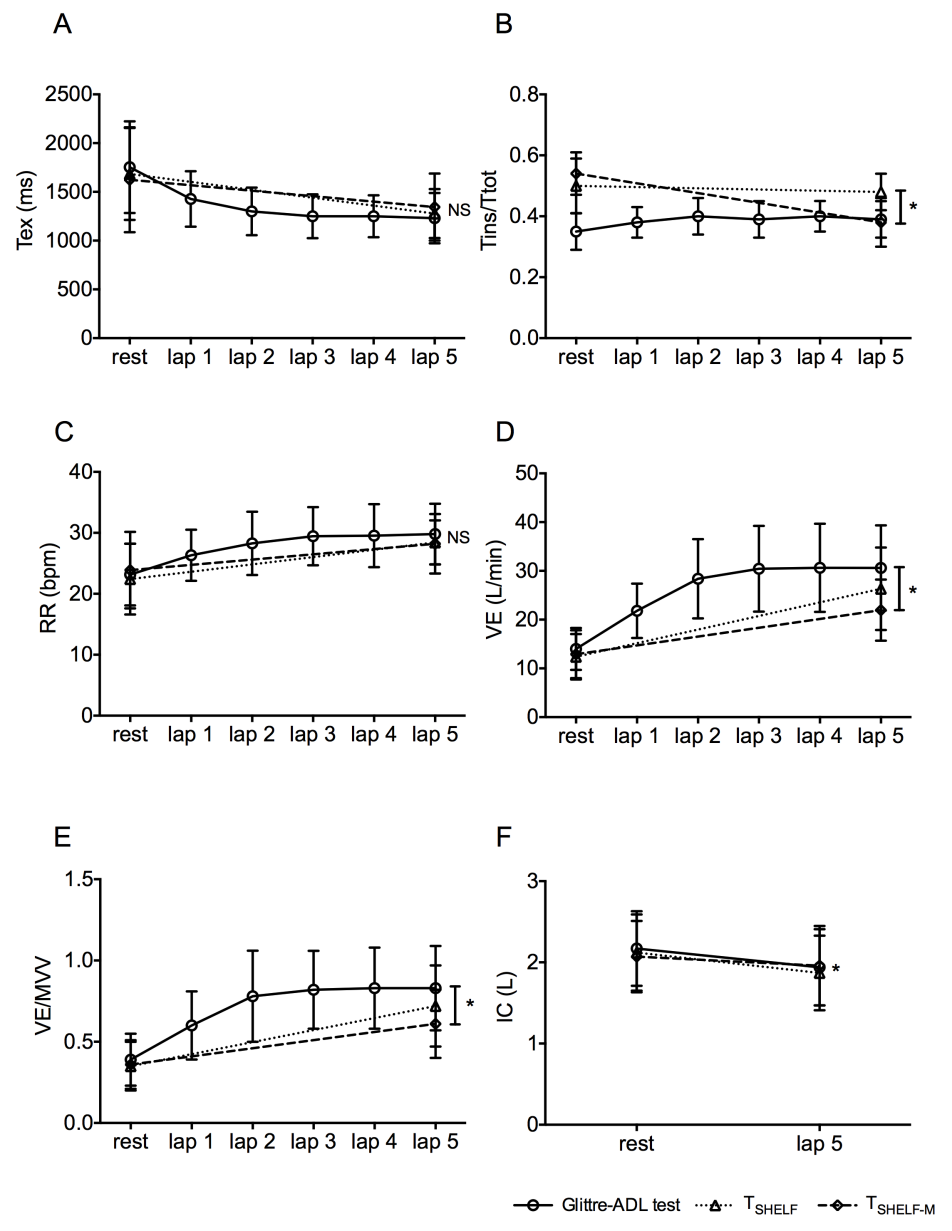
Comparison of the variation of the ventilatory behavior of the COPD group on every lap of the Glittre-ADL test (open circles and solid line) and the beginning and end of the T_SHELF_ (open triangles and dotted line) and T_SHELF-M_ (open diamonds and dashed line). Average data and standard deviation: (A) expiratory time; (B) inspiratory time/total time; (C) respiratory rate; (D) minute ventilation; (E) ventilatory demand; (F) inspiratory capacity; NS: not significant; *: p<0.05; NS * and refers to the ANOVA variation (Δ) between tests.

The mean differences in ∆VE/MVV between the Glittre-ADL test and the T_SHELF_ in the COPD and control groups were 6.52% (95% CI 0.03-13.0; p=0.51) and 9.64% (95% CI 6.37-12.9; p=0.001), respectively. The ∆VE/MVV in the T_SHELF-M_ was 12.4% lower than the T_SHELF_ in the COPDG (95% CI 7.56-17.3; p=0.007) and 7.42% lower in the control group (95% CI 4.54-10.3; p=0.001). Despite this, the time spent in the T_SHELF_ and T_SHELF-M_ was not significantly different in either group (p=0.81 for COPDG; p=0.51 for control group). The time spent in the T_SHELF_ and VE/MVV corresponded to 50.2% (95% CI 41.6-58.8) and 86.7% (95% CI 80.4 – 93.0) of the Glittre-ADL test in the COPDG. Regardless of the fact that COPD patients completed the T_SHELF_ faster than the Glittre-ADL test, changes in ventilatory variables were similar between them. Significant changes were only seen in the inspiratory-to-total cycle time ratio (p=0.04) and minute ventilation (p=0.03). Comparisons between tests for each group are presented in Table 2 and Figure 3.

### Dynamic hyperinflation (DH)

The inspiratory capacity decreased significantly at the end of the Glittre-ADL test (0.22±0.16L; p=0.001), T_SHELF_ (0.24±0.20L; p=0.001), and T_SHELF-M_ (0.11±0.16L; p=0.004) in the COPDG, while no statistically significant differences were observed in the inspiratory capacity of the control group (p=0.12). There were no statistical changes in the magnitude of DH caused by the Glittre-ADL test and T_SHELF_ in the COPDG (p=0.67). However, the DH caused by the T_SHELF-M_ was significantly lower than the T_SHELF_ (p=0.03).

In the COPDG, 15 subjects presented DH in the Glittre-ADL test, 17 in the T_SHELF_, and eight in the T_SHELF-M_; 12 patients with COPD had DH in the Glittre-ADL test and T_SHELF_ and seven patients in the Glittre-ADL test and T_SHELF-M_. Only three patients did not present DH in any of the tests. The performance of the control group in the tests was better than the COPDG (p=0.001 for all).

## Discussion

The main finding of this study was that the Glittre-ADL test and T_SHELF_ induced similar ventilatory demand and DH in the COPD patients, despite the shorter time to complete the T_SHELF_. When the upper extremity tasks were isolated in the T_SHELF-M_, the ventilatory responses were reduced.

Upper limb tasks often cause DH in COPD patients, with subsequent decrease in inspiratory capacity, increase in ventilatory effort, and overload of accessory respiratory muscles, in addition to higher levels of VO_2_ ^21^. In this study, we attempted to isolate the upper extremity tasks performed during the Glittre-ADL test as it has been suggested that they could explain the limitation of the test, especially in severely ill patients^8^. Recently, when analyzing the physiological responses of the Glittre-ADL test, Karloh et al.^9^ observed that, after the third lap, the oxygen consumption and minute ventilation reached a plateau similarly to the response usually observed after the third minute in the 6-minute walk test^9^. In the present study, the ventilatory variables showed a similar pattern in the COPD group, but it happened later in the control group (between the fourth and the last lap).

COPD patients have reduced ventilatory reserve and are unable to increase ventilation in response to increasing metabolic demand, even in activities performed in short periods. This may be explained by the limited inspiratory reserve volume^4^ as patients achieved almost 80% of MVV on the third lap of the test. Moreover, in the COPDG, the expiratory time decreased considerably until the third lap, generating an increase in the final expiratory reserve volume and contributing to DH.

DH occurs independently of disease severity^22^ and there is speculation that the presence and level of DH changes according to exercise type. Approximately 60% of COPD patients develop DH during upper limb activity, while a small proportion develops it while performing lower limb exercise with the same metabolic demand^7^. Since moving objects on and off shelves requires unsupported upper and lower limb movement, it is important to clarify that modifying the original Glittre-ADL test protocol, rather than the T_SHELF-M,_ helped us to understand the real role of the upper limbs during the test. The magnitude of the DH was lower when isolated upper limb activity was performed during the T_SHELF-M_ than when it was combined with other tasks such as squatting and bending down, both in the COPDG and in the control group. The comparisons between the different shelf tasks lead us to hypothesize that there might be other tasks that generate as much ventilatory demand as upper limb activity. It has been shown that postural muscle activities are altered with increased respiratory demand in healthy individuals^23^. Balance in the mediolateral direction depends on hip and trunk movements and decreases after upper limb activity in COPD patients. This could be related to the competition between the contribution of muscles to balance and ventilatory function^24^. Additionally, the squatting performed during the T_SHELF_ requires lower limb muscle activity, leading to higher energy consumption and metabolic and ventilatory demand in a shorter time^7^ compared to activities performed with upper limbs. Therefore, squatting and bending down could have contributed to higher DH in the T_SHELF_.

The COPDG achieved ventilatory values close to the MVV during the Glittre-ADL test and T_SHELF_, while in the control group, the VE/MVV during the Glittre-ADL test did not reach 40%. These results are similar to other published data showing that COPD patients almost reach their maximum ventilatory capacity during exercise and ADL^9^ ^,^ ^25^. COPD patients had a statistically lower variation in VE/MVV in the T_SHELF-M_ compared to the T_SHELF,_ although the times taken to perform tests were not different (p>0.05). Furthermore, the T_SHELF_ led to a VE/MVV that reached approximately 80% of the VE/MVV achieved during the Glittre-ADL test. Based on these findings, it could be speculated that the tasks performed with the shelf unit might be responsible for a greater increase in ventilatory demand during the Glittre-ADL test; however, this requires further investigation. A study comparing isolated activities based on the Glittre-ADL test, lasting one minute each, verified that walking up/down stairs required greater energy expenditure and resulted in more dyspnea and fatigue in COPD patients. In contrast, moving objects among shelves with unsupported arms led to a higher energy demand compared to the other tasks performed^26^. As our study aimed to compare different activities and not the effects of each activity on energy expenditure during the Glittre-ADL test, some remarks should be made. The present study demonstrated that approximately 50% of the time required to complete the Glittre-ADL test was spent on tasks in front of the shelf unit, as shown in a previous study^9^. Therefore, the comparison of different activities of this test with the same duration would not properly reflect the individual contribution of each task on energy expenditure and ventilatory and metabolic demand induced by the Glittre-ADL test.

Some limitations can be pointed out. First, static lung volumes such as residual volume and total lung capacity were not measured. This could have provided more data regarding pulmonary function in COPD patients. However, the focus of the present study was the dynamic change in lung volumes, therefore the appropriate methods were applied. The analysis of inspiratory capacity changes performed with slow vital capacity maneuver reveals changes in residual volume with accuracy and reproducibility^19^. Furthermore, patients used a short-acting bronchodilator prior to the tests, which could have influenced the magnitude of the DH. However, similar conditions were established for all patients during all tests. Another potential limitation was that the results could not be applied to long-term oxygen patients as they were excluded from the present study. The coupling of an external oxygen source would have changed the cardiorespiratory response results. Therefore, the course of these variables in patients with severe gas exchange impairment remains unknown. The power for the main findings of this study was 80% for the difference in DH between the T_SHELF_ and T_SHELF-M_ (p=0.03) and 70% for the difference in DH between the Glittre-ADL test and T_SHELF_ (p=0.67) in the COPDG.

This was the first study to analyze in detail the components of ventilatory response of the Glittre-ADL test and one specific activity performed during the test. The findings add more knowledge and value to the Glittre-ADL test, which causes greater VE/MVV in COPD patients than in healthy subjects during ADL. It also reinforces the advantage of its use in the clinical setting for prescribing pulmonary rehabilitation programs and evaluation of the programs’ results. The findings of this study contribute to a better understanding of the ventilatory mechanisms in the Glittre-ADL test. The results demonstrated that in COPD patients, ventilatory constraints could occur in simple tasks, such as moving upper limbs or squatting and bending down. The findings highlight the need to implement strategies that minimize the ventilatory demand during ADL, consequently breaking the vicious circle of inactivity, deconditioning, and dyspnea and preventing COPD progression and worsening. Future studies are necessary to compare effectively the metabolic, cardiovascular, and ventilatory responses of the four components of the Glittre-ADL test in patients with different levels of functional impairment.

## Conclusions

In summary, the Glittre-ADL test and the isolated shelf task from its circuit cause higher ventilatory demand and DH in patients with COPD compared to healthy subjects, but both tests were similar in COPD patients. However, when the activity with the upper limbs was isolated in the T_SHELF-M_, the ventilatory response was reduced in COPD patients. Our findings suggest that the shelf tasks of the Glittre-ADL test are probably responsible for the ventilatory overload of the test and that the squatting and bending down movements, commonly performed in ADL could explain the patients’ limitations observed during the test.
